# Structural instability of IκB kinase β promotes autophagic degradation through enhancement of Keap1 binding

**DOI:** 10.1371/journal.pone.0203978

**Published:** 2018-11-30

**Authors:** Mayu Kanamoto, Yoshihiro Tsuchiya, Yuki Nakao, Takafumi Suzuki, Hozumi Motohashi, Masayuki Yamamoto, Hideaki Kamata

**Affiliations:** 1 Department of Molecular Medical Science, Graduate School of Biomedical Science, Hiroshima University, Hiroshima, Japan; 2 Department of Dermatology, Mazuda Hospital, Hiroshima, Japan; 3 Department of Nutrition and Health Promotion, Hiroshima Jogakuin University, Hiroshima, Japan; 4 Department of Medical Biochemistry, Tohoku University Graduate School of Medicine, Sendai, Japan; 5 Department of Gene Expression Regulation, Institute of Development, Aging and Cancer, Tohoku University, Sendai, Japan; Lewis Katz School of Medicine at Temple University, UNITED STATES

## Abstract

IKKβ, an essential kinase of NF-κB signaling, is composed of an N-terminal kinase domain (KD) and a C-terminal scaffolding domain, containing a ubiquitin-like domain (ULD). The Hsp90 chaperon has special responsibility for folding of protein kinases including IKKβ. Here, we found that Hsp90 inhibition induced IKKβ degradation, which is partially mediated by Keap1. Geldanamycin (GA), a Hsp90 inhibitor, enhances association of IKKβ with Keap1 through the binding site in KD, and translocates IKKβ to detergent-insoluble fractions leading to its autophagic degradation. An electrophile tBHQ suppressed Keap1-mediated proteasomal Nrf2 degradation but not autophagic IKKβ degradation. Substitution mutation of Leu353 to Ala in the ULD destabilizes IKKβ, enhances its association with Keap1, translocates it to detergent-insoluble fractions, and causes its autophagic degradation. These results suggest that Keap1 is involved in the degradation of structural destabilized IKKβ and negative regulation of NF-κB under proteotoxic stress.

## Introduction

NF-κB is a critical transcription factor, regulating oxidative stress response, inflammation and carcinogenesis [1, 2]. In resting cells, NF-κB is inactivated by association with inhibitory proteins such as IκBα that prevent the DNA binding and nuclear transport. A vast array of stimuli including inflammatory cytokines and cellular stress activate NF-κB through the IκB kinase (IKK) complex, which consists of two catalytic subunits, IKKα and IKKβ, and a regulatory subunit NEMO/IKKγ [3–6]. While IKKβ mediates activation of the canonical NF-κB pathway in response to pro-inflammatory stimuli, IKKα has an indispensable role in non-canonical NF-κB signaling by phosphorylating NF-κB2 [7]. IKKβ is composed of kinase domain (KD), ubiquitin-like domain (ULD), scaffold/dimerization domain (SDD), and NEMO-binding domain (NBD). Crystal structure analysis showed that IKKβ is activated by trans auto-phosphorylation of Ser171 and Ser181 in the activation loop of KD through higher order oligomerization [8, 9]. IKKβ phosphorylates IκBα at N-terminal serines, Ser32 and Ser36, which leads to its ubiquitination and proteasomal degradation. These reactions result in nuclear translocation of NF-κB and binding of its cognate κB sites in the promoters of target genes. The NF-κB signaling pathways are associated with many human diseases, including inflammatory disease and cancer, which suggests that IKKβ can be a potential therapeutic target [10].

Maintenance of proteome integrity and protein homeostasis depend on a complex network of molecular chaperones, including conserved heat shock proteins (HSPs) [11, 12]. Environmental insults perturb cellular proteome homeostasis, triggering the proteotoxic stress response, or heat shock response, that includes the induction of expression genes encoding HSPs. HSPs are classified into several groups based on their molecular weight. Hsp90, Hsp70, and Hsp60, refer to families of HSPs on the order of 90, 70, and 60 kilodaltons in size, respectively. Hsp90, one of the most conserved HSPs, is essential not only in the protective heat shock response but also due to its chaperoning function in folding client proteins to their active conformations [13]. The Hsp90 chaperon is responsible for folding of protein kinases, and Hsp90 inhibitors such as geldanamycin (GA), stimulate kinase degradation [14]. It has been reported that Hsp90 is essential for IKKβ activity [15, 16], and Hsp90 inhibition results in IKKβ degradation [15–17]. Degradation of IKKβ is mediated by two major pathways that degrade cellular proteins in cells: the ubiquitin–proteasome system (UPS) and autophagy [17–19].

Keap1 is a ubiquitin ligase which interacts with a transcription factor Nrf2, a master regulator of the antioxidant response [20, 21]. Under quiescent conditions, Nrf2 is anchored in the cytoplasm by binding to Keap1, which in turn facilitates its proteolysis by UPS [22]. Oxidative stress and electrophiles disrupt the Keap1-Nrf2 complex, dissociating Nrf2 from Keap1 and translocating it into the nucleus where it can act as a transcription factor for series of antioxidant genes to defend against oxidative stress. Keap1 also binds to IKKβ and causes its degradation through UPS [18] or autophagy [23]. Recent studies have revealed a functional connection between IKKβ/NF-κB and Keap1/Nrf2 signaling pathways, which modulates inflammatory and carcinogenic processes.

Inhibition of HSPs or heat shock leads to proteotoxic stress that causes cellular protein damage or conformational changes such as protein misfolding, resulting in protein degradation. IKKβ degradation caused by Hsp90 inhibition may reflect an important adaptive cellular response which links proteotoxic stress response to inflammatory stimuli. However, the mechanism of IKKβ degradation by Hsp90 inhibition is yet be studied. In this study, we investigated the mechanism by which Hsp90 inhibition causes IKKβ degradation and found that structural destabilization of IKKβ enhances its binding to Keap1 and promotes its autophagic degradation by different mechanism from that of Nrf2.

## Materials and method

### Reagents and antibody

Anti-tubulin (H-300) and anti-HA (Y-11) rabbit antibodies were obtained from Santa Cruz Biotechnology. Anti-HA (11867423) rat monoclonal antibody and anti-Keap1 (10503-2-AP) rabbit antibody were purchased from Roche and Proteintech, respectively. Anti-IKKβ (2684), anti-phospho-IKKβ (2697) and anti-phospho-IκBα (2859) rabbit antibodies were procured from Cell Signaling. Anti-Flag (M2) mouse antibody was obtained from Sigma. MG132, Chloroquine, GA, 3-methyladenine(3-MA), and cycloheximide (CHX) were procured from Sigma-Aldrich. Tertiary Butylhydroquinone (tBHQ) was purchased from Tokyokasei.

### Cell culture, plasmid transfection, and luciferase assay

Human embryonic kidney (HEK293) and mouse embryonic fibroblast (MEF) cells were cultured in Dulbecco’s modified Eagle’s medium supplemented with 10% fetal bovine serum, 2 mM L-glutamine, 100 U/ml penicillin G, and 100 μg/ml streptomycin. HEK293 cells were obtained from RIKEN cell bank. WT and *Keap1*^*-/-*^ MEFs were prepared in the laboratory of Masayuki Yamamoto. Keap1 and Nrf2 were amplified from a human cDNA library by polymerase chain reaction (PCR), and cDNAs were cloned in expression vectors encoding HA and Flag sequences. Expression plasmids encoding IKKβ, IKKα, NEMO/IKKγ and IκBα were described previously [24]. Substitution mutants were generated by PCR. Plasmids were transfected into fibroblasts, which were cultured in Opti-MEM (Invitrogen) using Lipofectamine Plus (Invitrogen), following the manufacturer’s instructions. For luciferase assay, cells were transfected with reporters encoding NF-κB binding sites (pNF-κB luciferase plasmids), or Nrf2 binding sites (pNQO1 luciferase plasmids), together with pRK-TK Renilla-luciferase control plasmids. Luciferase activity was measured using Dual-Luciferase Reporter Assay System (Promega) following the manufacturer’s instructions.

### Cell fractionation

Cells were solubilized in buffer A consisting of 20 mM Tris-Cl (pH 7.5), 150 mM NaCl, 10 mM EGTA, 10 mM MgCl_2_, 60 mM β-glycerophosphate, 1 mM Na_3_VO_4_, 1 mM 4-amidino phenyl methyl sulfonyl fluoride, 50 KIU/ml aprotinin, 20 μg/ml pepstatin, 20 μg/ml leupeptin, 2 mM dithiothreitol and 1% Triton X-100. After centrifugation at 16,000 x *g* for 20 min at 4°C, the lysates were fractionated into supernatants and pellets. The pellets were then solubilized in buffer B containing 100 mM Tris-Cl, pH 6.8, 2% SDS, 5% glycerol, and 2.5% 2-mercaptoethanol. Cells solubilized in buffer B were used as total cell lysates.

### Immunoprecipitation and immunoblotting

For the immunoprecipitation assay of transfected cells, cell supernatants were incubated with anti-Flag (M2) Sepharose (Sigma), or with an antibody combined with Protein A and Protein G Sepharose (GE Healthcare) at 4°C. For the immunoprecipitation assay of endogenous proteins, cell lysates were incubated with an antibody together with TrueBlot anti-mouse or anti-rabbit IP beads (eBioscience) and subjected to immunoblotting using TrueBlot HRP-conjugated anti-mouse or anti-rabbit IgG antibodies (eBioscience). Proteins separated by gel electrophoresis were transferred to polyvinylidene difluoride membranes (Millipore) by an electroblotting apparatus (Mighty Small Transphor; Amersham) and subjected to immunoblotting using HRP-conjugated anti-mouse or anti-rabbit IgG antibodies (GE Healthcare). Antigen-antibody complexes were detected using SuperSignal West Pico Chemiluminescence System (Pierce).

### Immunostaining of cells

HEK293 cells (0.5 x 10^6^ cells) were transfected with 0.2 ug plasmids encoding HA-IKKβ, HA-IKKβ (L353A), Flag-Keap1, and GFP-LC3. Cells were grown on collagen-coated coverslips. After 18 h, cells were fixed with 4% paraformaldehyde in PBS for 30 min, and then fixed cells were permeabilized with 0.1% Triton X-100 in PBS for 30 min at room temperature. After washing with PBS, cells were stained with anti-HA rat monoclonal antibodies or anti-Flag (M2) mouse antibodies in Can Get Signal immunostain Immunoreaction Enhancer Solution (TOYOBO) for 2 h. After washing with PBS, coverslips were incubated with Cy3-conjugated anti-mouse IgG antibodies or FITC-conjugated anti-rat IgG antibodies (Jackson ImmunoResearch Laboratories) for 2 h and were observed under BZ-9000 Fluorescence Microscope (KEYENCE).

### Statistics

Data were expressed as means   standard deviation (S.D.) from at least three independent experiments and analyzed by two-tailed t-tests. Differences were considered statistically significant if at * P < 0.05, ** P < 0.01.

## Results

### Inhibition of Hsp90 promotes Keap1-mediated autophagic degradation of IKKβ through enhancing Keap1 binding

**Fig 1:**
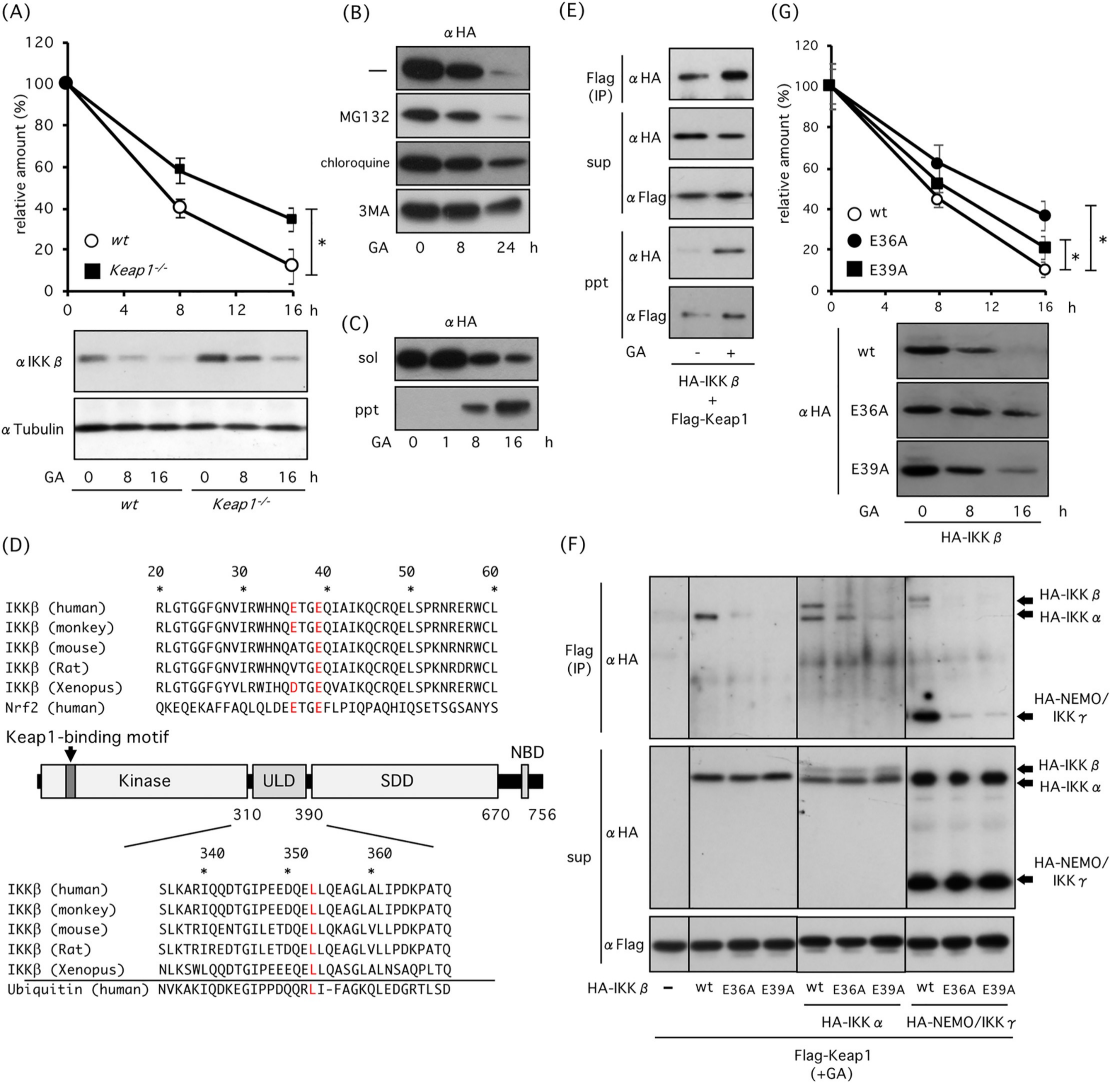
Effect of Hsp90 inhibition on IKKβ degradation. (A) Role of Keap1 in GA-induced IKKβ degradation. WT and *Keap1*^*-/-*^ MEFs were incubated with 2 μM GA, and expression levels of IKKβ were investigated. (B) Role of autophagy in GA-induced IKKβ degradation. HEK293 cells were transfected with HA-IKKβ and incubated for 18 h. Then cells were treated with GA in the presence or absence of 20 μM MG132, 20 μM chloroquine, and 4 mM 3MA. (C) Role of GA in IKKβ translocation into detergent soluble or insoluble fractions in HA-IKKβ-transfected HEK293 cells. (D) Schematic representation of IKKβ. Keap1-binding ETGE motif of IKKβ is located in the KD. (E, F) Role of E36 and E39 glutamate residues of IKKβ ETGE motif in GA-enhanced IKKβ-Keap1 association. After 18 h transfection of HEK293 cells with Flag-Keap1 and HA-IKKβ (E) or with HA-IKKβ, HA-IKKα and HA-NEMO/IKKγ (F), HEK293 cells were treated with GA for 4 h and then proteins were immunoprecipitated with anti-Flag agarose. (G) Involvement of E36 and E39 in GA-induced degradation. After 18 h transfection of HEK293 cells with HA-IKKβ mutants, cells were treated with GA. Statistically significance at * P < 0.05, ** P < 0.01.

When cells were treated with GA, the decrease in IKKβ levels was slower in *Keap1*^*-/-*^ MEF cells than in their wild-type counterparts, suggesting that Keap1 is partially involved in GA-induced IKKβ degradation (Fig 1A). Inhibitors of autophagy such as chloroquine and 3-MA, but not proteasome inhibitor MG132, delayed GA-induced degradation, indicating that autophagy mainly mediates GA-induced IKKβ degradation (Fig 1B). Misfolded proteins are known to form detergent-insoluble aggresomes. GA induces translocation of transfected HA-IKKβ into detergent-insoluble fractions (Fig 1C). IKKβ is composed of KD, ULD, SDD, and NBD, and the Keap1 binding motif which includes two essential glutamate residues (E36 and E39) locates in the N-terminal of KD (Fig 1D). Immunoprecipitation assay revealed that GA increases binding of IKKβ to Keap1 (Fig 1E). Glu-to-Ala substitution mutations at E36 and E39 (E36A and E39A) decreased GA-mediated enhanced IKKβ-Keap1 association (Fig 1F). Besides, these mutations reduced the binding of co-expressed IKKα and NEMO/IKKγ to Keap1. GA-mediated degradation of IKKβ mutants (E36A and E39A) were slower than that of wild type IKKβ (wt IKKβ), suggesting that inhibition of Hsp90 promotes autophagic degradation of IKKβ mediated in part by binding to Keap1.

### Keap1-mediated IKKβ degradation is not inhibited by tBHQ

**Fig 2:**
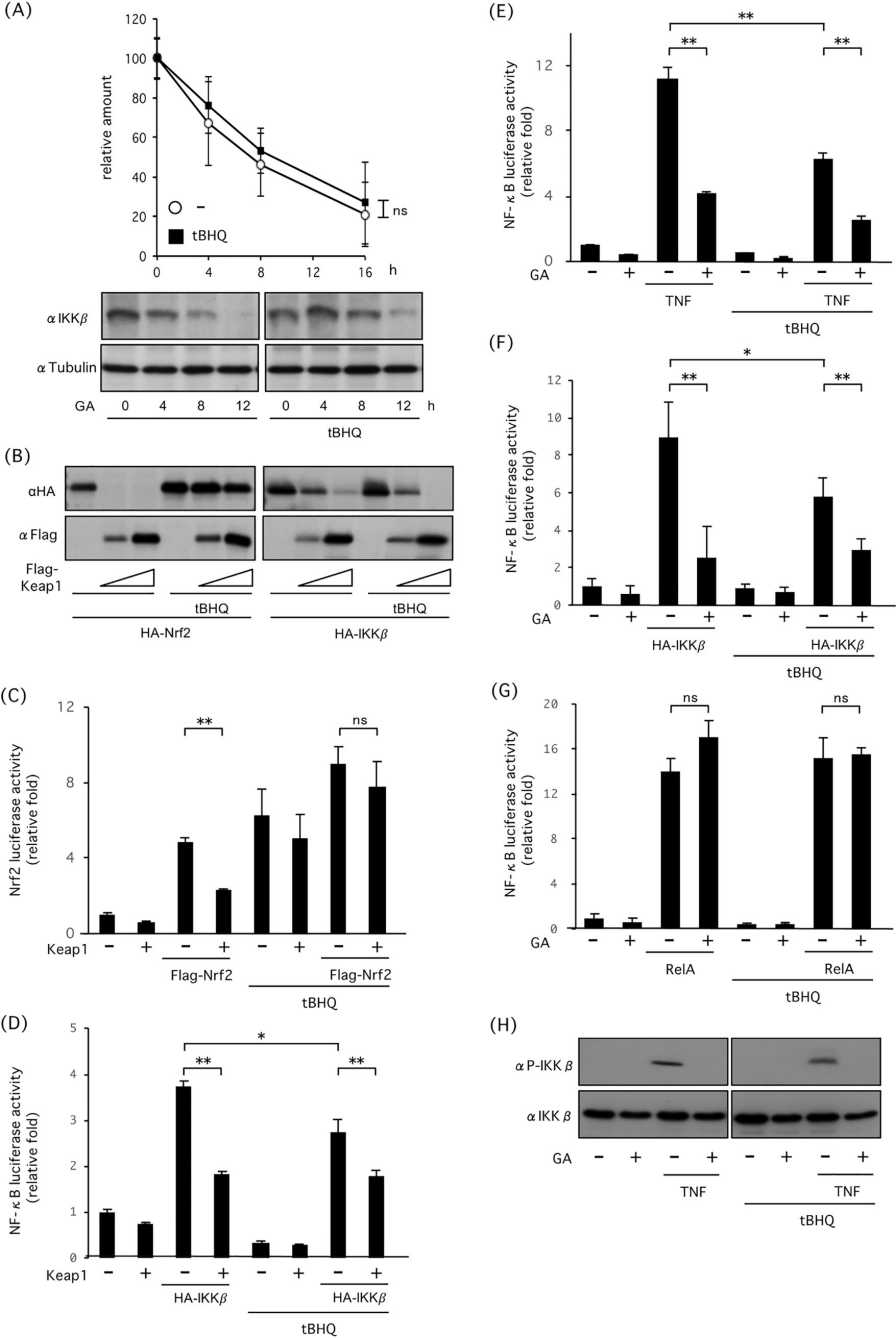
Inhibitory effect of tBHQ on Keap1-mediated IKKβ degradation. (A) Inhibitory effect of tBHQ on GA-mediated IKKβ degradation. Cells were treated with GA in the presence or absence of 50 μM tBHQ. (B) Inhibitory effect of tBHQ on Keap1-mediated Nrf2 or/and IKKβ degradation. HEK293 cells were transfected with HA-IKKβ, HA-Nrf2 and Flag-Keap1, and incubated with or without tBHQ for 18 h. (C) Nrf2 luciferase activity of HEK293 cells transfected with Nrf2-reporter together with Flag-Keap1 and HA-Nrf2 in the presence or absence of tBHQ. (D) NF-κB luciferase activity of HEK293 cells transfected with NF-κB-reporter together with Flag-Keap1 and HA-IKKβ in the presence or absence of tBHQ. Both luciferase activities were measured 18 h post-transfection. (E) NF-κB luciferase activity of HEK293 cells transfected with NF-κB reporter, and incubated with or without GA, tBHQ, and 50 ng/ml TNFα (as an inflammation stimulator) for 18 h. (F, G) NF-κB luciferase activity of HEK293 cells transfected with NF-κB reporter together with or without HA-IKKβ(F) or Flag-RelA (G), incubated in presence or absence of GA and tBHQ for 18 h. (H) Inhibitory effect of tBHQ on GA-mediated IKKβ inactivation and degradation. HEK293 cells were treated with GA for 6 h and then stimulated with TNFα for 20 min. * P < 0.05, ** P < 0.01.

Cysteine residues in Keap1 are modified by electrophiles, such as tBHQ, or reactive oxygen species, leading to Keap1 dissociation from Nrf2. As a result, Nrf2 is stabilized and it subsequently translocates to the nucleus. To elucidate whether tBHQ stabilizes IKKβ, we investigated degradation of IKKβ in the presence or absence of tBHQ, in cells treated with GA, and found that tBHQ did not prevent GA-induced IKKβ degradation (Fig 2A). Co-transfection assay revealed that tBHQ prevented Keap1-induced Nrf2 degradation, but not IKKβ degradation (Fig 2B). tBHQ promoted Nrf2 activity and prevented Keap1-mediated suppression of Nrf2 (Fig 2C). However, it did not inhibit Keap1-mediated suppression of IKKβ-induced NF-κB activation (Fig 2D). Treatment of cells with GA suppressed TNFα-induced NF-κB activation, and tBHQ did not prevent GA-mediated NF-κB suppression (Fig 2E). Co-transfection with IKKβ significantly activated NF-κB, and GA suppressed IKKβ-induced NF-κB activation (Fig 2F). GA-mediated NF-κB suppression was not prevented by tBHQ in IKKβ-transfected cells. However, GA did not suppress NF-κB-activation in cells transfected with RelA, suggesting that GA specifically suppressed IKKβ activity (Fig 2G). Western blot analysis revealed that GA suppressed TNF-induced IKKβ phosphorylation, and tBHQ did not inhibit GA-mediated IKKβ suppression (Fig 2H). These results indicate that Hsp90 inhibition leads to Keap1-mediated autophagic degradation that is not inhibited by tBHQ.

### L353A mutation destabilizes IKKβ and leads its autophagic degradation

**Fig 3:**
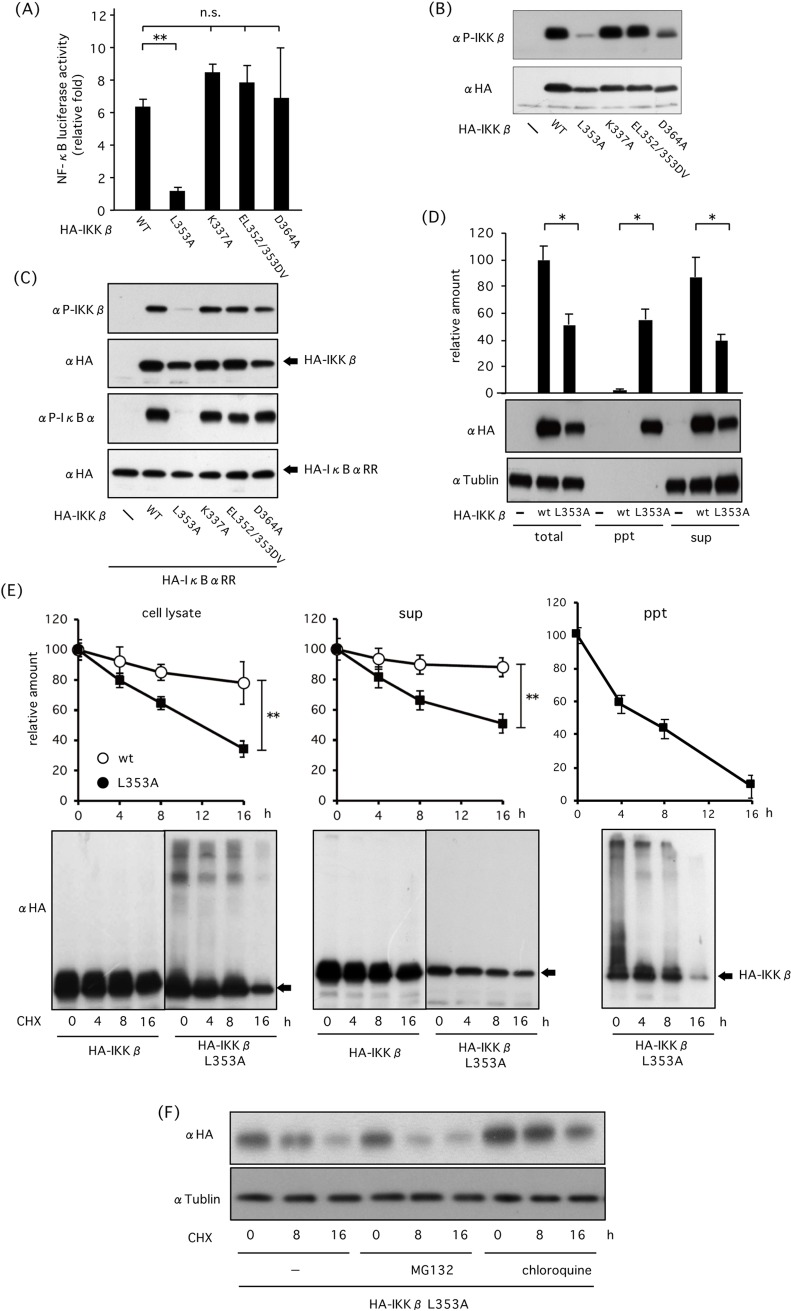
Effect of L353A mutation in the ULD of IKKβ on structural stability or degradation of IKKβ. (A) Effect of different HA-IKKβ mutations on IKKβ-induced NF-κB activation in terms of NF-κB luciferase activity. HEK293 cells were transfected with NF-κB reporter and HA-IKKβ mutants. After 18 h, luciferase activities were measured. (B) Effect of HA-IKKβ mutants on IKKβ autophosphorylation. HEK293 cells were transfected with HA-IKKβ mutants and phosphorylation of IKKβ was detected by western blot analysis. (C) Effect of HA-IKKβ on IKKβ-induced IκBα phosphorylation. HEK293 cells were transfected with HA-IKKβ mutants together with HA-IκBαRR, and phosphorylation of IκBα was detected by western blot analysis. (D) Role of L353A mutation in HA-IKKβ translocation into detergent insoluble or insoluble fractions of HEK293 cells. Cells were transfected with wild type or mutated HA-IKKβ(L353A), and expression levels were investigated in total cell lysates, detergent soluble and insoluble fractions. (E) Effect of L353A mutation on IKKβ degradation in different fractions. HEK293 cells were transfected with wild type or mutated HA-KKβ(L353A) mutant. After incubation with 10 μg/ml CHX, expression levels were investigated in total cell lysates, detergent soluble and insoluble fractions. (F) Autophagy mediates degradation of L353A mutant. HEK293 cells transfected with HA-IKKβ or HA- KKβ(L353A) mutants were incubated with CHX in the presence or absence of MG132 and chloroquine. * P < 0.05, ** P < 0.01.

Structural destabilized mutant of IKKβ was studied to elucidate whether structural destabilization per se induces autophagic degradation. The ULD interacts with KD and SDD in a highly organized manner that is required for a critical interaction with IκBα and kinase activity [8]. We generated several Ala substitution mutants in the ULD and found that Leu-to-Ala substitution mutation at Leu353 abolishes IKKβ-induced NF-κB activation (Fig 3A), which was consistent with previous reports [8, 25]. Moreover, autophosphorylation was reduced in L353A mutant (Fig 3B). Co-transfection of HEK293 cells with HA-IKKβ mutants and ubiquitination-resistant HA-IκBαRR mutant revealed that L353A mutant hardly phosphorylate IκBα (Fig 3C). L353A mutant was localized to the detergent-insoluble fraction (Fig 3D). L353A mutant degraded more rapidly than wt IKKβ, and the rate of disappearance was faster in the detergent-insoluble fraction than in the detergent-soluble fraction (Fig 3E). Degradation of L353A mutant was partially prevented by chloroquine but not by MG132, suggesting that autophagy mainly mediates degradation of structural destabilized L353A mutant (Fig 3F).

### L353A mutation increases Keap1 binding

**Fig 4:**
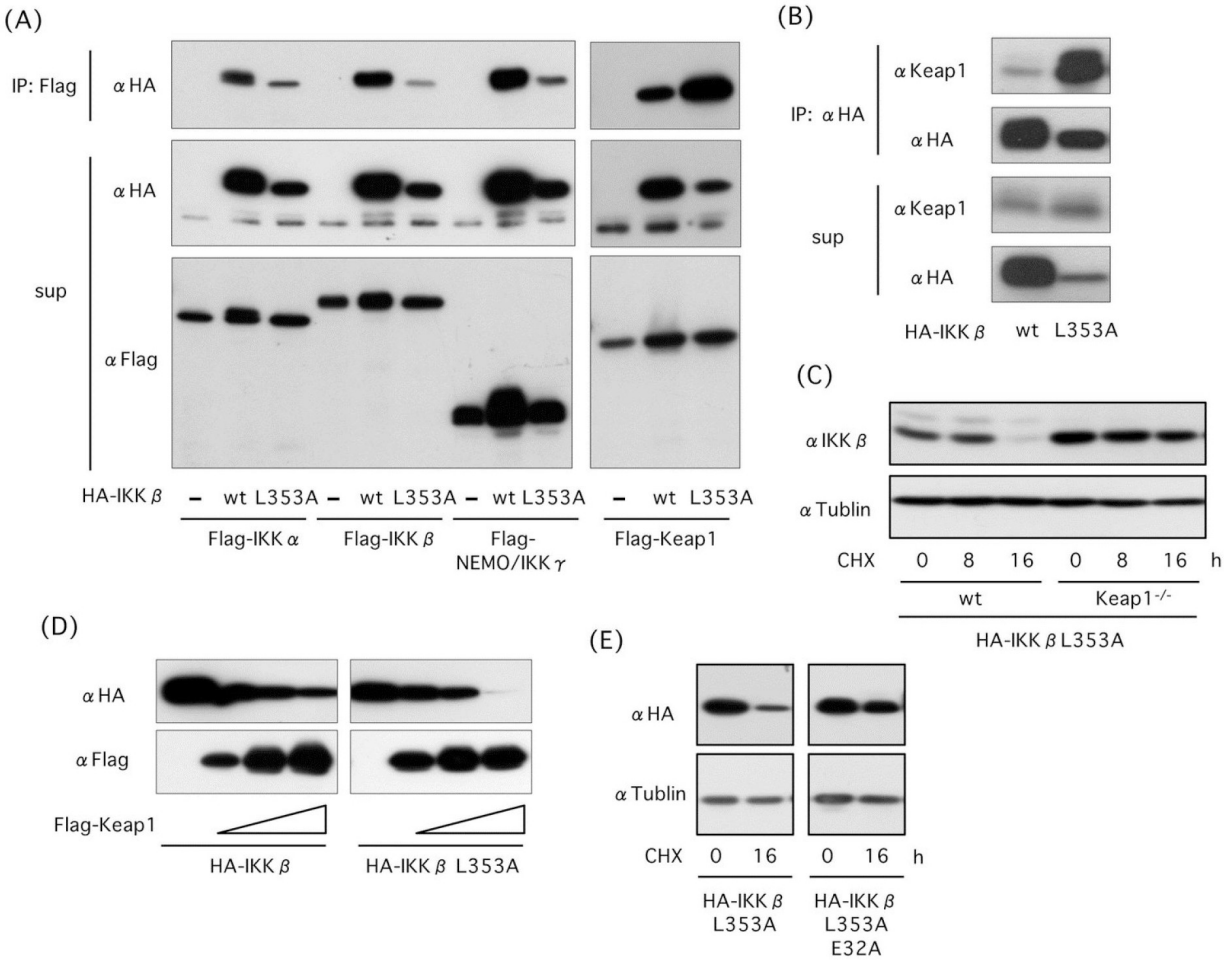
Effect of L353A mutation on association of IKKβ with Keap1 and autophagic degradation. (A) Effect of L353A mutation on IKKβ-Keap1 binding. Cells were transfected with wild type or mutated HA-IKKβ(L353A) together with Flag-IKKα, Flag-IKKβ, Flag-NEMO/IKKγ, and Flag-Keap1. After 18 h, cell lysates were immunoprecipitated with an anti-Flag agarose beads, and association of IKKβ with IKKα, IKKβ, NEMO/IKKγ, and Keap1 was investigated. (B) Effect of L353A mutation on association of IKKβ with endogenous Keap1. HEK293 cells transfected with HA-IKKβ or HA-IKKβ(L353A) were immunoprecipitated with an anti-HA antibody. (C) Role of Keap1 in IKKβ(L353A) degradation. After transfection of WT and *Keap1*^*-/-*^ MEF cells with mutated HA-IKKβ(L353A), cells were treated with CHX and degradation of HA- KKβ(L353A) was investigated by western blot analysis. (D) Effect of L353A mutation on Keap1-mediated IKKβ degradation. HEK293 cells were transfected with HA-IKKβ or HA-IKKβ(L353A) together with Flag-Keap1, and expression levels were investigated in cell lysates by western blot analysis. (E) Involvement of Keap1-binding motif in degradation of L353A mutant. After 18 h transfection with HA-IKKβ(L353A) and double mutant HA-IKKβ (L353A/E36A), HEK293 cells were incubated with CHX. Expression of transfected IKKβ proteins was detected by western blot with an anti-HA antibody.

To elucidate whether Keap1 is involved in degradation of L353A mutant, we investigated the interaction of Keap1 to L353A mutant. Immunoprecipitation assay of transfected cells revealed that binding of Keap1 to IKKβ is increased in L353A mutant, whereas binding of IKKα, IKKβ and NEMO/IKKγ to IKKβ were not changed (Fig 4A). Additionally, the increased interaction of endogenous Keap1 to L353A mutant was detected in cells (Fig 4B). The reduction in the amount of L353A mutant was slower in *Keap1*^*-/-*^ than in wt MEFs under CHX treatment, suggesting that Keap1 is involved in L353A degradation (Fig 4C). Co-transfection assay revealed that Keap1 degraded L353A mutant more effectively than wt IKKβ (Fig 4D). We generated expression plasmids encoding double mutants of L353A/E36A and L353A/E39A. L353A/E36A was expressed effectively, whereas L353A/E39A was hardly expressed in cells. Then we compared degradation ratio of L353A/E36A to L353A and found that L353A/E36A is more stable than L353A (Fig 4E). These results indicate that structural destabilization of IKKβ increases its interaction to Keap1, leading to autophagic degradation.

### Keap1 is subjected to autophagic degradation

**Fig 5:**
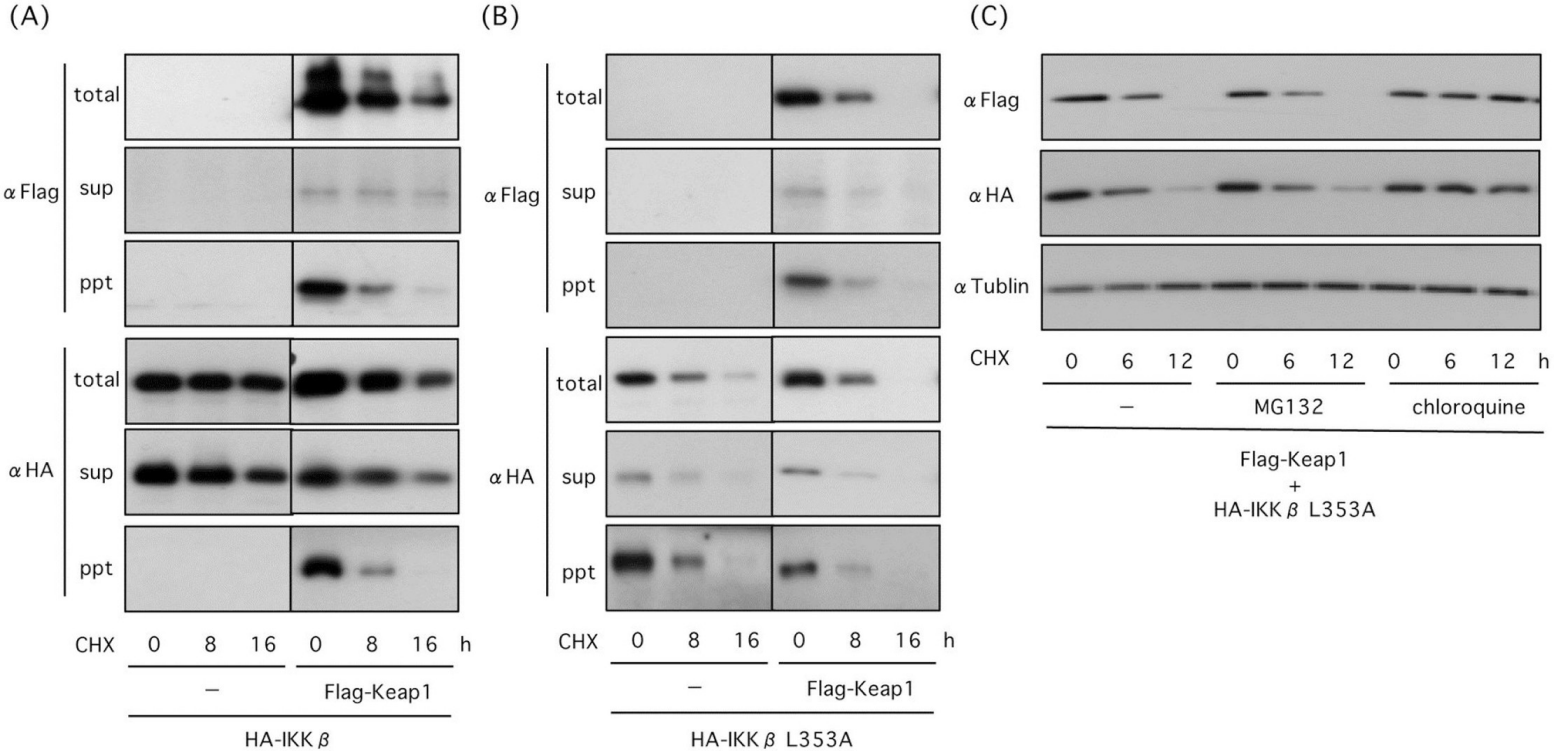
Keap1 is degraded with IKKβ by autophagy. (A) Expression of Keap1 promoted IKKβ degradation and Keap1 is degraded concomitant with IKKβ. HEK293 cells were transfected with wild type HA-KKβ and Flag-Keap1. After 18 h of transfection, 10 μg/ml CHX was added to cell culture. Then cell lysates were prepared following incubation for 8 and 16 h. Expression levels of proteins were investigated in total cell lysates, detergent soluble and insoluble fractions. (B) Keap1 was degraded concomitant with IKKβ(L353A) mutant. HEK293 cells were transfected with HA- IKKβ(L353A) together with Flag-Keap1 and were treated with CHX. (C) Keap1 was degraded by autophagy. HEK293 cells transfected with Flag-Keap1 and HA- KKβ(L353A) were incubated with CHX in the presence or absence of MG132 and chloroquine.

Keap1 is degraded by autophagy in response to oxidative stress [26, 27]. To elucidate whether Keap1 is degraded with IKKβ, we transfected cells with plasmids encoding wild type or mutated HA-IKKβ(L353A) together with or without Flag-Keap1. Co-expression of Keap1 induced translocation of wild type IKKβ into the detergent-insoluble fraction and its degradation, and Keap1 is degraded concomitant with IKKβ (Fig 5A). Similarly, Keap1 is degraded concomitant with HA-IKKβ(L353A) when cells were co-transfected with Flag-Keap1 and HA-IKKβ(L353A) (Fig 5B). Degradation of Keap1 was suppressed by chloroquine, but not by MG132, in cells co-transfected with HA-IKKβ(L353A) (Fig 5C). These results suggest that Keap1 is degraded concomitant with IKKβ by autophagy.

### Structural destabilized IKKβ colocalizes with autophagic machinery proteins in cells

**Fig 6:**
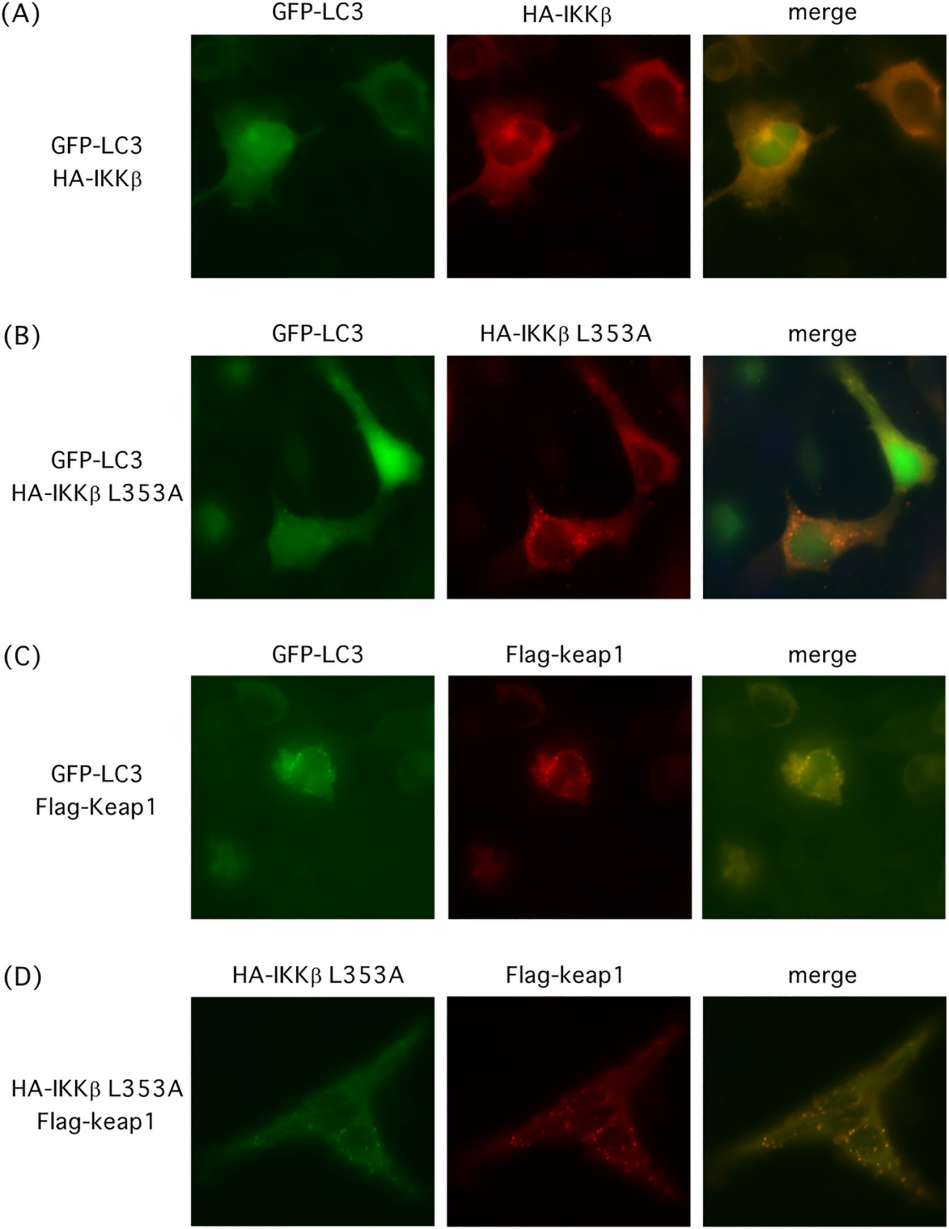
Colocalization of L353A mutant of IKKβ with LC3 and Keap1 in the cytoplasm. (A) HA-IKKβ was dispersed in the cytoplasm. HEK293 cells were transfected with plasmids encoding wild type HA-IKKβ and GFP-LC3. (B) HA-IKKβ(L353A) was colocalized with GFP-LC3 in small punctuate structures. HEK293 cells were transfected with HA-IKKβ(L353A) and GFP-LC3. (C) Flg-Keap1 was colocalized with GFP-LC3 in small punctuate structures. HEK293 cells were transfected with Flg-Keap1 and GFP-LC3. (D) HA-IKKβ(L353A) was colocalized with Flg-Keap1 in small punctuate structures. HEK293 cells were transfected with HA-IKKβ(L353A) and Flg-Keap1. Following transfection, cells were incubated for 18 h and subjected to immunostaining.

Atg8/LC3 protein plays an essential role in autophagosome maturation and is widely used as an autophagosome marker. To confirm the role of autophagic/lysosomal machinery in degradation of structural destabilized IKKβ, we investigated cellular localization of IKKβ, Keap1, and autophagosome. HA-IKKβ appeared to be dispersed throughout the cytoplasm (Fig 6A). Whereas, although HA-IKKβ(L353A) was dispersed throughout the cytoplasm, some portion of HA-IKKβ(L353A) proteins formed small punctuate structures and colocalized with GFP-LC3 in the cytoplasm (Fig 6B). The majority of Flag-Keap1 proteins was dispersed throughout the cytoplasm, and some portion of Flag-Keap1 formed small punctuate structures and colocalized with GFP-LC3 (Fig 6C). HA-IKKβ(L353A) and Flag-Keap1 were colocalized in the small punctuate structures in the cytoplasm (Fig 6D). These results suggest that structural destabilized IKKβ interacts with Keap1 in the autophagic machinery protein complex and is degraded by autophagy.

## Discussion

The IKKβ/NF-κB and Keap1/Nrf2 signaling pathways play an essential role in cellular signaling in response to inflammatory stimuli and oxidative stress. It has been reported that activation of NF-κB antagonizes Nrf2-mediated transcription by either inhibition of Nrf2-DNA binding [28] or depriving CREB binding protein (CBP) from Nrf2 and facilitating the interaction of recruited histone deacetylase 3 (HDAC3) at the Nrf2-DNA binding site [29]. Nrf2-deficient MEFs show greater activation of NF-κB in response to lipopolysaccharide (LPS) stimulus [30], and Nrf2 suppresses NF-κB-induced transcriptional upregulation of proinflammatory cytokines, including IL-6 and IL-1β in macrophages [31]. Indeed, NF-κB activation is suppressed in tBHQ-treated cells in which Nrf2 is activated (Fig 2D, 2E and 2F). Thus, cellular responses to inflammatory stimuli and oxidative stress is regulated by crosstalk between the IKKβ/NF-κB signaling and the Keap1/Nrf2 signaling pathways.

Although Keap1 is involved in structurally destabilized IKKβ degradation, the contribution of Keap1 to degradation is partial; thus, other factors would be involved in IKKβ degradation as well. It has been reported that Drosophila NEMO/IKKγ has an LC3-interacting region (LIR) or Atg8-interacting motif (AIM) in the N-terminal region which recruits the IKK complex to the autophagosome, and acts as a selective autophagy receptor that mediates the degradation of the IKK complex [32]. Whereas human NEMO/IKKγ lacks functional LIR/AIM and does not as a selective autophagy receptor for degradation of the IKK complex. Instead, NEMO/IKKγ is ubiquitinated in human and could also interact with selective autophagy receptors via ubiquitin [33, 34]. In this study, we found that structural destabilized IKKβ interacts with Keap1 and is degraded by autophagy. Thus, several mechanisms would be involved in degradation of the IKK complex in cells under proteotoxic stress.

Cells are challenged by various extrinsic and intrinsic insults which provoke proteotoxic stress under conditions of injury, inflammation, and oxidative stress. As disturbance of proteome homeostasis is associated with a broad range of human diseases, maintenance of proteome integrity is essential to maintain cellular fitness and functions. To protect cells from proteotoxic stress, cells mobilize heat shock proteins including Hsp90 that unfold proteins and proteolysis systems that remove damaged proteins. In this study, it was found that Hsp90 inhibition, which causes proteotoxic stress, enhances interaction of IKKβ with Keap1. These findings also suggest that cells respond to proteotoxic stress by regulating IKKβ/NF-κB and Keap1/Nrf2 signaling pathways. The Keap1/Nrf2 and IKKβ/NF-κB signaling pathways have interesting features concerning the proteolytic mechanisms such as autophagy and UPS. Keap1 suppresses Nrf2 activation by UPS, whereas it is degraded by autophagy in response to oxidative stress [26, 27]. IKKβ induces NF-κB activation through degradation of IκBα by UPS, whereas it is degraded by autophagy in response to proteotoxic stress. Furthermore, IKKβ contributes to the induction of autophagy in an NF-κB-independent manner [35], and Keap1 interacts with p62, a selective autophagy receptor, which binds to Atg8/LC3 protein, resulting in autophagy [36, 37]. There is a plausibility that oxidative stress and proteotoxic stress induce selective degradation of Keap1 and IKKβ by autophagy and provoke specific cellular responses.

## References

[1] Ben-Neriah Y, Karin M. Inflammation meets cancer, with NF-kappaB as the matchmaker. Nat Immunol. 2011;12(8):715–23. 10.1038/ni.2060 . 21772280 10.1038/ni.2060

[2] Hayden MS, Ghosh S. NF-kappaB, the first quarter-century: remarkable progress and outstanding questions. Genes Dev. 2012;26(3):203–34. 10.1101/gad.183434.111 ; PubMed Central PMCID: PMCPMC3278889. 22302935 10.1101/gad.183434.111 PMC3278889

[3] DiDonato JA, Hayakawa M, Rothwarf DM, Zandi E, Karin M. A cytokine-responsive IkappaB kinase that activates the transcription factor NF-kappaB. Nature. 1997;388(6642):548–54. Epub 1997/08/07. 10.1038/41493 . 9252186 10.1038/41493

[4] Mercurio F, Zhu H, Murray BW, Shevchenko A, Bennett BL, Li J, et al IKK-1 and IKK-2: cytokine-activated IkappaB kinases essential for NF-kappaB activation. Science. 1997;278(5339):860–6. Epub 1997/11/05. . 9346484 10.1126/science.278.5339.860

[5] Woronicz JD, Gao X, Cao Z, Rothe M, Goeddel DV. IkappaB kinase-beta: NF-kappaB activation and complex formation with IkappaB kinase-alpha and NIK. Science. 1997;278(5339):866–9. Epub 1997/11/05. . 9346485 10.1126/science.278.5339.866

[6] Yamaoka S, Courtois G, Bessia C, Whiteside ST, Weil R, Agou F, et al Complementation cloning of NEMO, a component of the IkappaB kinase complex essential for NF-kappaB activation. Cell. 1998;93(7):1231–40. Epub 1998/07/10. . 9657155 10.1016/s0092-8674(00)81466-x

[7] Scheidereit C. IkappaB kinase complexes: gateways to NF-kappaB activation and transcription. Oncogene. 2006;25(51):6685–705. Epub 2006/10/31. 10.1038/sj.onc.1209934 . 17072322 10.1038/sj.onc.1209934

[8] Xu G, Lo YC, Li Q, Napolitano G, Wu X, Jiang X, et al Crystal structure of inhibitor of kappaB kinase beta. Nature. 2011;472(7343):325–30. 10.1038/nature09853 ; PubMed Central PMCID: PMC3081413. 21423167 10.1038/nature09853 PMC3081413

[9] Polley S, Huang DB, Hauenstein AV, Fusco AJ, Zhong X, Vu D, et al A structural basis for IkappaB kinase 2 activation via oligomerization-dependent trans auto-phosphorylation. PLoS biology. 2013;11(6):e1001581 10.1371/journal.pbio.1001581 ; PubMed Central PMCID: PMC3678999. 23776406 10.1371/journal.pbio.1001581 PMC3678999

[10] Karin M. Nuclear factor-kappaB in cancer development and progression. Nature. 2006;441(7092):431–6. Epub 2006/05/26. 10.1038/nature04870 . 16724054 10.1038/nature04870

[11] Kim YE, Hipp MS, Bracher A, Hayer-Hartl M, Hartl FU. Molecular chaperone functions in protein folding and proteostasis. Annu Rev Biochem. 2013;82:323–55. Epub 2013/06/12. 10.1146/annurev-biochem-060208-092442 . 23746257 10.1146/annurev-biochem-060208-092442

[12] Saibil H. Chaperone machines for protein folding, unfolding and disaggregation. Nat Rev Mol Cell Biol. 2013;14(10):630–42. Epub 2013/09/13. 10.1038/nrm3658 ; PubMed Central PMCID: PMCPMC4340576. 24026055 10.1038/nrm3658 PMC4340576

[13] Taipale M, Jarosz DF, Lindquist S. HSP90 at the hub of protein homeostasis: emerging mechanistic insights. Nat Rev Mol Cell Biol. 2010;11(7):515–28. Epub 2010/06/10. 10.1038/nrm2918 . 20531426 10.1038/nrm2918

[14] Caplan AJ, Mandal AK, Theodoraki MA. Molecular chaperones and protein kinase quality control. Trends Cell Biol. 2007;17(2):87–92. 10.1016/j.tcb.2006.12.002 . 17184992 10.1016/j.tcb.2006.12.002

[15] Chen G, Cao P, Goeddel DV. TNF-induced recruitment and activation of the IKK complex require Cdc37 and Hsp90. Mol Cell. 2002;9(2):401–10. . 11864612 10.1016/s1097-2765(02)00450-1

[16] Broemer M, Krappmann D, Scheidereit C. Requirement of Hsp90 activity for IkappaB kinase (IKK) biosynthesis and for constitutive and inducible IKK and NF-kappaB activation. Oncogene. 2004;23(31):5378–86. Epub 2004/04/13. 10.1038/sj.onc.1207705 . 15077173 10.1038/sj.onc.1207705

[17] Qing G, Yan P, Xiao G. Hsp90 inhibition results in autophagy-mediated proteasome-independent degradation of IkappaB kinase (IKK). Cell Res. 2006;16(11):895–901. 10.1038/sj.cr.7310109 . 17088896 10.1038/sj.cr.7310109

[18] Lee DF, Kuo HP, Liu M, Chou CK, Xia W, Du Y, et al KEAP1 E3 ligase-mediated downregulation of NF-kappaB signaling by targeting IKKbeta. Mol Cell. 2009;36(1):131–40. 10.1016/j.molcel.2009.07.025 ; PubMed Central PMCID: PMCPMC2770835. 19818716 10.1016/j.molcel.2009.07.025 PMC2770835

[19] Niida M, Tanaka M, Kamitani T. Downregulation of active IKK beta by Ro52-mediated autophagy. Mol Immunol. 2010;47(14):2378–87. Epub 2010/07/16. 10.1016/j.molimm.2010.05.004 ; PubMed Central PMCID: PMCPMC2918734. 20627395 10.1016/j.molimm.2010.05.004 PMC2918734

[20] Hirotsu Y, Katsuoka F, Funayama R, Nagashima T, Nishida Y, Nakayama K, et al Nrf2-MafG heterodimers contribute globally to antioxidant and metabolic networks. Nucleic Acids Res. 2012;40(20):10228–39. Epub 2012/09/12. 10.1093/nar/gks827 ; PubMed Central PMCID: PMCPMC3488259. 22965115 10.1093/nar/gks827 PMC3488259

[21] Itoh K, Wakabayashi N, Katoh Y, Ishii T, Igarashi K, Engel JD, et al Keap1 represses nuclear activation of antioxidant responsive elements by Nrf2 through binding to the amino-terminal Neh2 domain. Genes Dev. 1999;13(1):76–86. Epub 1999/01/14. ; PubMed Central PMCID: PMCPMC316370. 9887101 10.1101/gad.13.1.76 PMC316370

[22] Suzuki T, Motohashi H, Yamamoto M. Toward clinical application of the Keap1-Nrf2 pathway. Trends Pharmacol Sci. 2013;34(6):340–6. 10.1016/j.tips.2013.04.005 . 23664668 10.1016/j.tips.2013.04.005

[23] Kim JE, You DJ, Lee C, Ahn C, Seong JY, Hwang JI. Suppression of NF-kappaB signaling by KEAP1 regulation of IKKbeta activity through autophagic degradation and inhibition of phosphorylation. Cell Signal. 2010;22(11):1645–54. 10.1016/j.cellsig.2010.06.004 . 20600852 10.1016/j.cellsig.2010.06.004

[24] Tsuchiya Y, Asano T, Nakayama K, Kato T Jr., Karin M, Kamata H. Nuclear IKKbeta is an adaptor protein for IkappaBalpha ubiquitination and degradation in UV-induced NF-kappaB activation. Mol Cell. 2010;39(4):570–82. Epub 2010/08/28. 10.1016/j.molcel.2010.07.030 . 20797629 10.1016/j.molcel.2010.07.030

[25] May MJ, Larsen SE, Shim JH, Madge LA, Ghosh S. A novel ubiquitin-like domain in IkappaB kinase beta is required for functional activity of the kinase. J Biol Chem. 2004;279(44):45528–39. 10.1074/jbc.M408579200 . 15319427 10.1074/jbc.M408579200

[26] Taguchi K, Fujikawa N, Komatsu M, Ishii T, Unno M, Akaike T, et al Keap1 degradation by autophagy for the maintenance of redox homeostasis. Proc Natl Acad Sci U S A. 2012;109(34):13561–6. Epub 2012/08/09. 10.1073/pnas.1121572109 ; PubMed Central PMCID: PMCPMC3427110. 22872865 10.1073/pnas.1121572109 PMC3427110

[27] Bae SH, Sung SH, Oh SY, Lim JM, Lee SK, Park YN, et al Sestrins activate Nrf2 by promoting p62-dependent autophagic degradation of Keap1 and prevent oxidative liver damage. Cell Metab. 2013;17(1):73–84. Epub 2013/01/01. 10.1016/j.cmet.2012.12.002 . 23274085 10.1016/j.cmet.2012.12.002

[28] Yu M, Li H, Liu Q, Liu F, Tang L, Li C, et al Nuclear factor p65 interacts with Keap1 to repress the Nrf2-ARE pathway. Cell Signal. 2011;23(5):883–92. 10.1016/j.cellsig.2011.01.014 . 21262351 10.1016/j.cellsig.2011.01.014

[29] Liu GH, Qu J, Shen X. NF-kappaB/p65 antagonizes Nrf2-ARE pathway by depriving CBP from Nrf2 and facilitating recruitment of HDAC3 to MafK. Biochim Biophys Acta. 2008;1783(5):713–27. 10.1016/j.bbamcr.2008.01.002 . 18241676 10.1016/j.bbamcr.2008.01.002

[30] Thimmulappa RK, Lee H, Rangasamy T, Reddy SP, Yamamoto M, Kensler TW, et al Nrf2 is a critical regulator of the innate immune response and survival during experimental sepsis. J Clin Invest. 2006;116(4):984–95. 10.1172/JCI25790 ; PubMed Central PMCID: PMCPMC1421348. 16585964 10.1172/JCI25790 PMC1421348

[31] Kobayashi EH, Suzuki T, Funayama R, Nagashima T, Hayashi M, Sekine H, et al Nrf2 suppresses macrophage inflammatory response by blocking proinflammatory cytokine transcription. Nat Commun. 2016;7:11624 10.1038/ncomms11624 ; PubMed Central PMCID: PMCPMC4879264. 27211851 10.1038/ncomms11624 PMC4879264

[32] Tusco R, Jacomin AC, Jain A, Penman BS, Larsen KB, Johansen T, et al Kenny mediates selective autophagic degradation of the IKK complex to control innate immune responses. Nat Commun. 2017;8(1):1264 Epub 2017/11/04. 10.1038/s41467-017-01287-9 ; PubMed Central PMCID: PMCPMC5668318. 29097655 10.1038/s41467-017-01287-9 PMC5668318

[33] Zotti T, Scudiero I, Settembre P, Ferravante A, Mazzone P, D'Andrea L, et al TRAF6-mediated ubiquitination of NEMO requires p62/sequestosome-1. Mol Immunol. 2014;58(1):27–31. Epub 2013/11/26. 10.1016/j.molimm.2013.10.015 ; PubMed Central PMCID: PMCPMC3909464. 24270048 10.1016/j.molimm.2013.10.015 PMC3909464

[34] Fliss PM, Jowers TP, Brinkmann MM, Holstermann B, Mack C, Dickinson P, et al Viral mediated redirection of NEMO/IKKgamma to autophagosomes curtails the inflammatory cascade. PLoS Pathog. 2012;8(2):e1002517 Epub 2012/02/10. 10.1371/journal.ppat.1002517 ; PubMed Central PMCID: PMCPMC3271075. 22319449 10.1371/journal.ppat.1002517 PMC3271075

[35] Criollo A, Senovilla L, Authier H, Maiuri MC, Morselli E, Vitale I, et al The IKK complex contributes to the induction of autophagy. The EMBO journal. 2010;29(3):619–31. 10.1038/emboj.2009.364 ; PubMed Central PMCID: PMC2830700. 19959994 10.1038/emboj.2009.364 PMC2830700

[36] Komatsu M, Kurokawa H, Waguri S, Taguchi K, Kobayashi A, Ichimura Y, et al The selective autophagy substrate p62 activates the stress responsive transcription factor Nrf2 through inactivation of Keap1. Nat Cell Biol. 2010;12(3):213–23. Epub 2010/02/23. 10.1038/ncb2021 . 20173742 10.1038/ncb2021

[37] Komatsu M, Waguri S, Koike M, Sou YS, Ueno T, Hara T, et al Homeostatic levels of p62 control cytoplasmic inclusion body formation in autophagy-deficient mice. Cell. 2007;131(6):1149–63. Epub 2007/12/18. 10.1016/j.cell.2007.10.035 . 18083104 10.1016/j.cell.2007.10.035

